# Aggressive Clinical Course and Malignant Transformation of a Meningeal Melanocytoma of the Pontomedullary Region: Diagnostic and Therapeutic Implications

**DOI:** 10.1055/a-2753-9601

**Published:** 2025-12-03

**Authors:** Oyku Ozturk, Mehmet A. Inan, Muammer M. Sahin, Emrah Celtikci

**Affiliations:** 1 Department of Neurosurgery, Gazi University Faculty of Medicine, Ankara, Türkiye; 2 Department of Pathology, Gazi University Faculty of Medicine, Ankara, Türkiye; 3 Department of Otorhinolaryngology/Head and Neck Surgery, Gazi University Faculty of Medicine, Ankara, Türkiye

**Keywords:** brainstem tumor, CNS melanoma, leptomeningeal tumors, malignant transformation, meningeal melanocytoma

## Abstract

**Background:**

Primary melanocytic tumors of the central nervous system (CNS) are rare neoplasms that range from benign melanocytomas to aggressive malignant melanomas. Although meningeal melanocytomas are generally considered indolent lesions, malignant transformation and distant metastasis can occur.

**Case Description:**

We report the case of a 33-year-old male with a bulbopontine meningeal melanocytoma who developed systemic metastases, culminating in a fatal outcome. Despite initial histopathologic features of benignity and absence of BRAF mutation, the lesion showed aggressive behavior.

**Conclusion:**

This case underscores the diagnostic pitfalls associated with primary CNS melanocytic tumors and highlights the importance of long-term vigilance, even for histologically benign lesions.

## Introduction

Primary melanocytic tumors of the central nervous system (CNS) are uncommon entities originating from leptomeningeal melanocytes, with an estimated incidence of 1 per 10 million annually. These tumors include a spectrum of lesions: benign (melanocytoma and diffuse melanocytosis) and malignant (melanoma and melanomatosis). 1 2 Although melanocytomas are typically slow-growing and noninvasive, their potential for malignant transformation remains poorly understood. Here, we present a case of a histopathologically benign brainstem meningeal melanocytoma with subsequent systemic metastasis, offering insights into its aggressive biological potential and emphasizing the diagnostic and prognostic uncertainty that often accompanies these lesions.

## Case Report

**Fig. 1:**
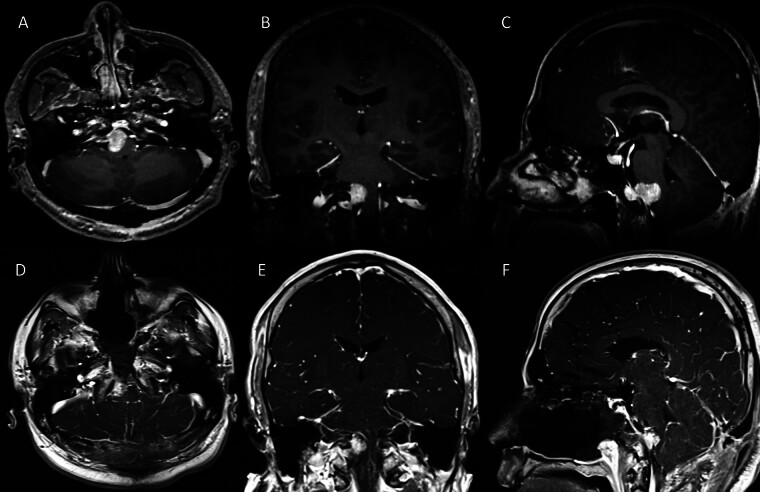
Preoperative axial ( **A** ), coronal ( **B** ), sagittal ( **C** ), and postoperative axial ( **D** ), coronal ( **E** ), sagittal ( **F** ) contrast-enhanced T1-weighted MRI demonstrating a hyperintense mass in the bulbopontine region.

A 33-year-old male presented with occipital headache, Valsalva-induced respiratory difficulty, and dysphagia. Neurological examination revealed bilateral nystagmus. Magnetic resonance imaging (MRI) revealed a T1-hyperintense, contrast-enhancing lesion centered in the right bulbopontine region, extending into the prepontine, premedullary, and right lateral cerebellomedullary cistern ( Fig. 1 ).

**Fig. 2:**
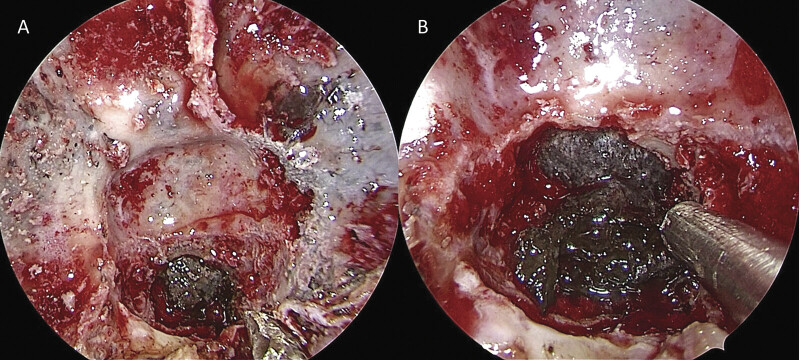
Intraoperative view of black–gray dural-based tumor invading skull base structures ( **A, B** ).

**Fig. 3:**
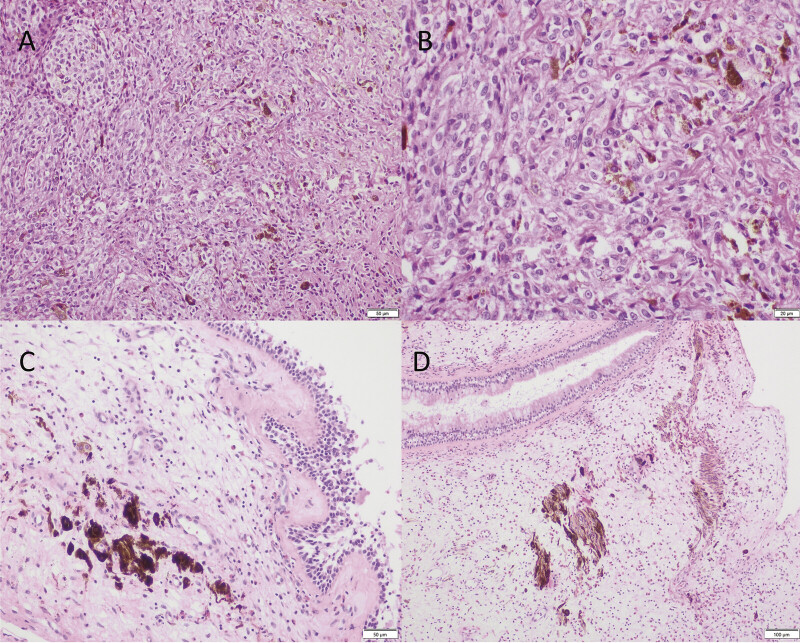
H&E stain of tumor cells showing melanin-rich spindle morphology with minimal atypia ( **A** , ×200; **B** , ×400). Benign melanosis in the sphenoid sinus ( **C** , ×200; **D** , ×100).

After the evaluation by a multidisciplinary council, surgery was planned as two sessions. During the first surgical procedure, suboccipital craniotomy with C1 laminectomy for decompression and tumoral mass excision with biopsy from the anterior part of the bulbus were performed. A biopsy sample was reported as benign melanosis on the bone. One month later, the second surgical procedure was performed by an endoscopic endonasal transsphenoidal approach for the resection. A firm, gray–black colored, dural-based mass invading the clival and sellar bones was identified and subtotally resected with the help of the neuronavigation 3 ( Fig. 2 ). Histopathology showed well-differentiated, melanin-rich spindle cells without mitotic activity, necrosis, or nuclear atypia—consistent with a diagnosis of meningeal melanocytoma ( Fig. 3 ). Additional biopsies from the sphenoid sinus and dura also revealed benign melanosis. Since it revealed the melanosis patient was referred to the departments of dermatology and ophthalmology for further evaluation. No skin and choroidal lesions were identified, and BRAF mutation testing was negative.

During follow-up, the patient developed hepatic lesions detected by abdominal computed tomography. A liver biopsy was done and confirmed metastatic melanoma with HMB45-MART1-Tyrosinase antibody positivity. The tests for BRAF mutation and PD-L1 expression were negative. Since the histopathological diagnosis was uncertain before surgery, systemic screening—including whole-body positron emission tomography (PET)—was not deemed necessary in the preoperative period. However, after the diagnosis of melanocytoma was made intraoperatively and the disease progressed, whole-body PET demonstrated widespread osseous metastases. Despite adjuvant radiotherapy, the patient died of progressive disease.

## Discussion

This case illustrates a paradoxical clinical course: a histologically benign meningeal melanocytoma with subsequent rapid systemic dissemination. Several hypotheses may explain this discrepancy:

1. Sampling error: histologic sections may not represent the entire lesion; focal malignant areas could have been missed.
2. Biological spectrum: melanocytomas may occupy a continuum with melanomas, especially in the CNS, where neural crest–derived melanocytes may harbor latent oncogenic potential.
3. Immunophenotypic silence: the absence of classic melanoma markers (e.g., BRAF mutation, mitosis) does not preclude aggressive behavior, as supported by recent molecular profiling studies. 4 5

Radiologically, both melanocytomas and melanomas demonstrate T1 hyperintensity and T2 hypointensity due to melanin's paramagnetic effects. However, radiology alone cannot reliably differentiate benign from malignant lesions. Furthermore, diffuse leptomeningeal melanocytosis and melanomatosis, though rare, may resemble infectious or inflammatory conditions on imaging and cerebrospinal fluid analysis. 6 7

No standardized treatment protocol exists for primary CNS melanocytic tumors. 8 Gross total resection remains the cornerstone of treatment, particularly for localized masses. In our case, complete resection was not feasible due to brainstem adherence. Although radiotherapy was administered postoperatively, it failed to prevent systemic spread. There is limited evidence regarding the efficacy of systemic therapies in primary CNS melanocytomas, particularly those undergoing malignant transformation.

This case raises two essential clinical considerations: (1) even histologically benign melanocytic CNS tumors warrant long-term surveillance; (2) absence of systemic disease at presentation does not rule out future metastasis.

## Conclusion

Meningeal melanocytomas, while typically benign, can exhibit aggressive biological behavior and metastasize. Long-term follow-up and a high index of suspicion are essential, even when histologic and molecular findings suggest indolence. This case emphasizes the need for multidisciplinary vigilance and may support broader resection and earlier adjunctive therapy in select patients.
